# Evolution of Fruit Traits in *Ficus* Subgenus *Sycomorus* (Moraceae): To What Extent Do Frugivores Determine Seed Dispersal Mode?

**DOI:** 10.1371/journal.pone.0038432

**Published:** 2012-06-05

**Authors:** Rhett D. Harrison, Nina Rønsted, Lei Xu, Jean-Yves Rasplus, Astrid Cruaud

**Affiliations:** 1 Key Laboratory of Tropical Forest Ecology, Xishuangbanna Tropical Botanical Garden, Chinese Academy of Science, Mengla, Yunnan, China; 2 Botanic Garden, Natural History Museum of Denmark, Copenhagen, Denmark; 3 INRA-UMR Centre de Biologie et de Gestion des Populations, CBGP (INRA/IRD/CIRAD/Montpellier SupAgro), Montferrier-sur Lez, France; Michigan State University, United States of America

## Abstract

Fig trees are a ubiquitous component of tropical rain forests and exhibit an enormous diversity of ecologies. Focusing on *Ficus* subgenus *Sycomorus*, a phenotypically diverse and ecologically important Old World lineage, we examined the evolution of fruit traits using a molecular phylogeny constructed using 5 kilobases of DNA sequence data from 63 species (50% of global diversity). In particular, we ask whether patterns of trait correlations are consistent with dispersal agents as the primary selective force shaping morphological diversity or if other ecological factors may provide a better explanation? Fig colour, size and placement (axial, cauliflorous, or geocarpic) were all highly evolutionarily liable, and the same fruit traits have evolved in different biogeographic regions with substantially different dispersal agents. After controlling for phylogenetic autocorrelation, we found that fig colour and size were significantly associated with fig placement and plant-life history traits (maximum plant height and leaf area, respectively). However, contrary to prevailing assumptions, fig placement correlated poorly with known dispersal agents and appears more likely determined by other factors, such as flowering phenology, nutrient economy, and habitat preference. Thus, plant life-history, both directly and through its influence on fig placement, appears to have played a prominent role in determining fruit traits in these figs.

## Introduction

Many plants co-opt animals to disperse their seeds by offering a reward for swallowing them in the form of fruit. Fruits vary tremendously in size and nature of the reward, and are presented in a variety of ways that require different abilities on the part of the seed disperser to locate and access them. The obvious correlation between the morphology and behaviour of putative seed dispersers and fruit characteristics often has led to an assumption that co-evolution with seed dispersers has driven the diversification of fruit syndromes [1], [2]. However, this hypothesis remains largely untested [2]. Moreover, frugivore assemblages are typically highly variable in space and time and comprise species ranging from seed predators to seed dispersers that vary substantially in body size and other traits affecting seed dispersal outcomes both qualitatively and quantitatively. Furthermore, the same lineage of plants, oftentimes the same species, may be exposed to unrelated frugivore lineages, with disparate morphologies and behaviour, in different parts of their range. We might, therefore, expect seed dispersal modes to be rather loosely defined [1], [3] and determined to a substantial degree by other aspects of plant ecology, such as nutrient economy or regeneration niche.

Figs (*Ficus*; Moraceae) comprise a large (ca. 750 species), pantropical woody plant genus that exhibits an enormous diversity of ecologies, including different plant life-forms, a huge range in plant size and leaf size, and a broad spectrum of fruit types [4]–[6]. However, all species possess a similar pollination system [7]. Highly specific fig pollinating wasps (Agaonidae, Chalcidoidea, Hymenoptera) enter a closed inflorescence (or “fig”) through a narrow, bract-lined passage to reproduce. Once inside, they pollinate and attempt to oviposit on some flowers. In monoecious *Ficus* species, ovules that receive an egg are galled and the wasp larva feeds on the gall tissue, while pollinated flowers missed by the wasps develop into seeds. Later when the adult wasp offspring emerge, they first mate within the fig and then the female wasps disperse carrying the fig’s pollen. Shortly thereafter the fig ripens into a fig fruit (pseudo-fruit) [6]. In dioecious species, the figs on male trees specialise in producing wasps (and thereby dispersing pollen), while those on female trees produce only seeds. The wasps enter the female figs and attempt to oviposit, but the styles are too long and they fail to lay any eggs [6]. In addition, there are large numbers of non-pollinating wasp (mostly Chalcidoidea) species that exploit the mutualism. Most of these use long ovipositors to pierce the thick wall of the fig from the outside and have a negative impact on pollinator production [8].

The combination of high global and local species richness and diverse ecologies built around a highly conserved reproductive system makes *Ficus* an ideal model for comparative study [9], [10]. However, previous evolutionary studies have been limited to datasets with either sparse sampling at a global level (e.g. [11]) or taxonomically diverse assemblages from a single locality [2], [12]. Phylogenetically focused studies offer a better opportunity to examine the evolution of functional traits and their interactions [13], [14].

**Figure 1:**
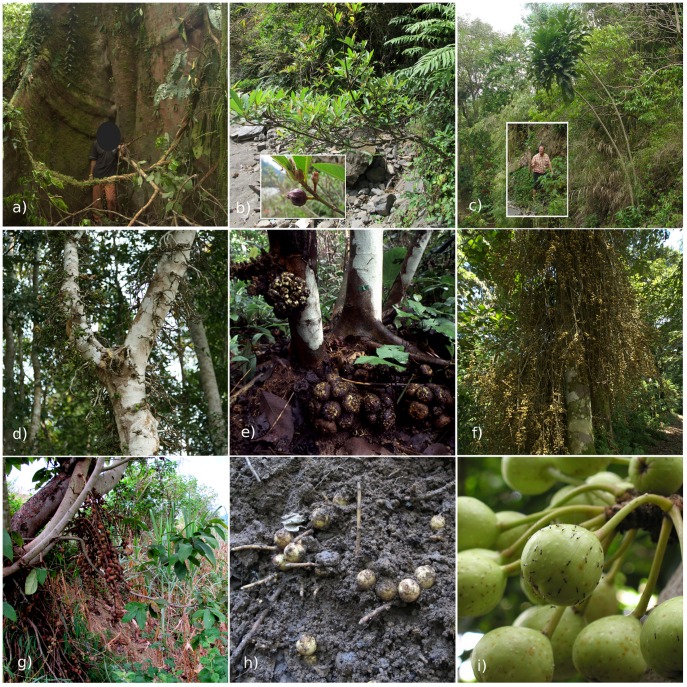
Diversity of functional traits in *Ficus* subgenus *Sycomorus*. a) *F. variegata* reaches up to 40 m high and is often one of the largest trees in secondary forests (man in photograph ∼ 1.5 m tall (face covered to protect identity)). The cauliflorous figs, borne from small nodes, can just be made out on the trunk. b) *F. squamosa*, rheophytic (river side) shrub up to 1.5 m high with axial figs (inset). c) *F. pseudopalma* (inset to scale, the man, who is ∼2 m tall, is holding up a dead leaf) has the second largest leaves in the subgenus and is one of only two monopodial (unbranched) species [15]. d) *F. hispida* has cauliflorous figs borne on woody branchlets (cauliflorus type (i)). e) *F. cereicarpa* is a cauliflorous species with very large figs (∼10 cm diameter). This is a male tree, which bears figs around the base of the tree, as is typical of several other species. Older figs, whose wasps have already emerged, can be seen rotting behind and under bunches of newer figs. f) *F. ribes* has small cauliflorous figs borne on rope-like stolons (cauliflorus type (ii)). *F. semicordata*: g) a female tree bearing figs at the base of the trunk and h) male figs buried in the soil. For the latter, the leaf litter and soil were scrapped away to reveal the figs. i) Male fig of *F. variegata* with non-pollinating wasps (*Sycophaga* sp.) ovipositing through the wall. The brown dots on other figs in the background are bruises resulting from earlier ovipositor insertions. Cauliflorous figs, like this, are often heavily attacked by non-pollinating wasps, which can significantly reduce pollinator production and thus pollen dispersal.

Here, we examine the evolution of fruit traits in *Ficus* subgenus *Sycomorus*, the most diverse subgenus. *Sycomorus* is monophyletic with approximately 130 species distributed from Africa to Fiji and is the only subgenus with both monoecious and dioecious members. The figs vary from ∼0.5 cm to >10 cm in diameter and are presented in a diversity of positions and styles (Fig. 1) [15]. Some species have axial figs (Fig. 1b). A large proportion are cauliflorous and may be borne from small nodes (Fig. 1a), on ramifying woody branchlets (Fig. 1d), or on long rope-like stolons that dangle from the trunk and main branches (Fig. 1f). In one particularly interesting form, the figs are borne on stolons that run along the ground for up to ten or more metres (Fig. 1g) [4]. In most of these geocarpic species the figs lie half-buried in the surface soil immediately beneath the leaf litter, but in some species they may be found up to 10 cm underground (Fig. 1h) [15]. How the pollinating wasps are able to enter such figs remains a mystery. The seeds of *Sycomorus* species may be dispersed by birds or mammals, including arboreal, terrestrial or scansorial species, and bats [16]. In stature species vary from wispy shrubs (Fig. 1b) less than one metre in height to canopy giants with trunks over a metre in diameter (Fig. 1a). Many species are pioneer plants and a critical component of regenerating forests [5]. In summary, *Sycomorus* is a species-rich, phenotypically diverse, widely distributed and ecologically important Old World lineage.

We employed multiple genetic loci (5 kb) to reconstruct the evolutionary history of *Sycomorus*. Next we examined the evolution of fig colour (red or green, see Methods section), size and placement (axial, cauliflorous types (i) and (ii), or geocarpic, see Methods section) with respect to other functional traits, including breeding system, maximum plant and leaf size, and biogeography. Specifically, we asked whether patterns of trait correlations are consistent with dispersal agents as the primary selective force shaping fruit morphological diversity or if other ecological factors might provide a better explanation.

## Results

### Phylogenetic Analyses

**Figure 2:**
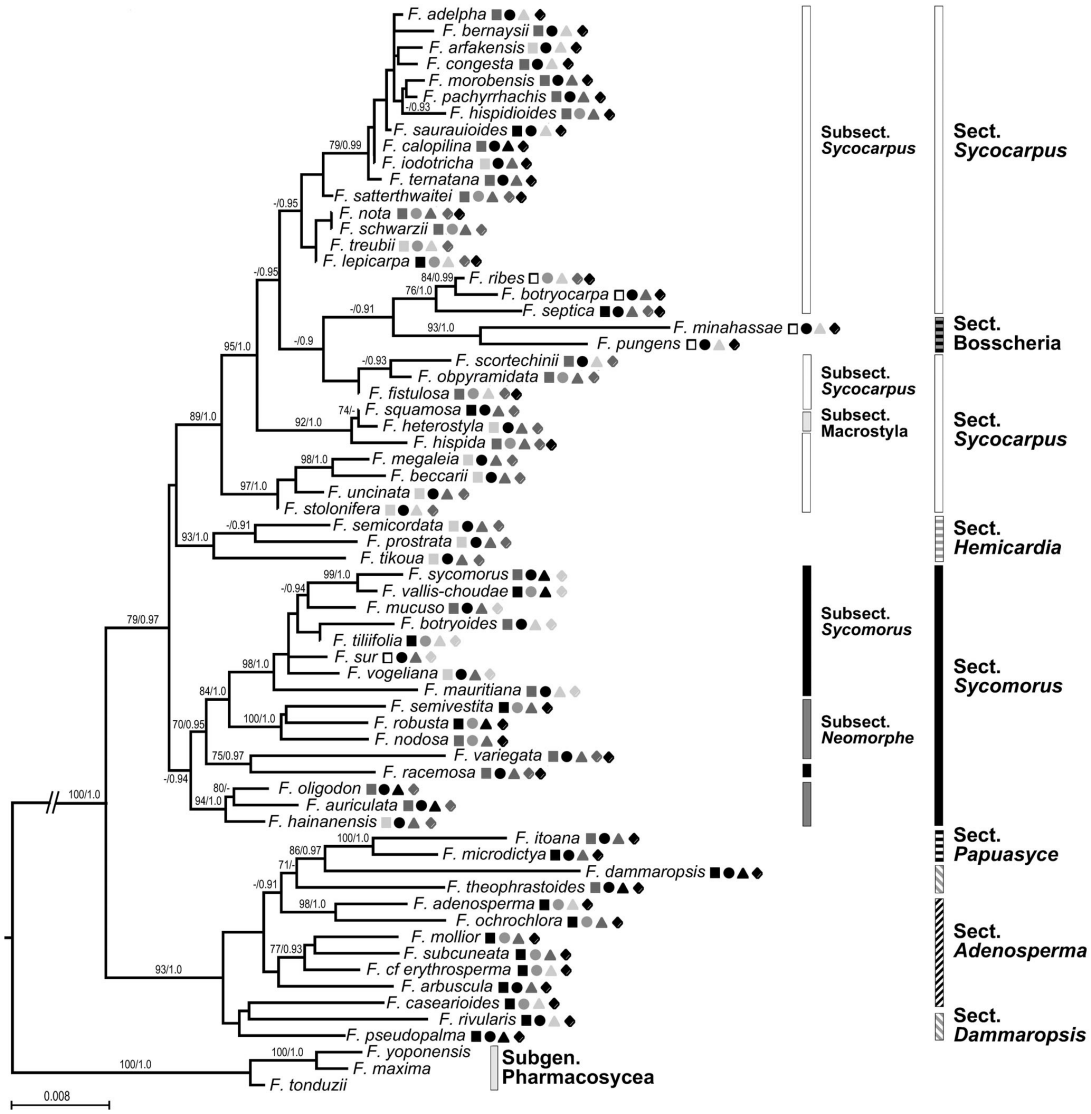
Phylogeny of *Ficus* subgenus *Sycomorus* using maximum likelihood estimation. The Bayesian topology was similar and we have mapped node support from both analyses (BP/PP). Node support was mapped for nodes with >75 BP or >0.90 PP. Also shown are fruit traits, including fig placement (squares; black = axial, dark grey = cauliflorous type (i), no fill = cauliflorous type (ii), light grey = geocarpic), fig colour (circles; black = red, dark grey = green), and fig diameter (triangles; light grey = <2 cm, dark grey = 2–<4 cm, black = 4+cm), biogeographic distribution (diamonds; light grey = Africa (+Madagascar and Indian Ocean), dark grey = Asia (West of Wallace’s line), black = Pacific (East of Wallace’s line)), and current taxonomy based on morphological characteristics.

The genes contained few gaps and these were easily aligned. All reconstructions produced similar topologies with moderate to strong supports for most clades. We arbitrarily chose to map node support values on the ML topology (Fig. 2). Our phylogenetic reconstruction corresponded poorly to current taxonomy, confirming results of other phylogenetic studies (e.g. [11]) (Fig. 2).

### Evolution of Fruit Traits

Fig colour was an evolutionarily liable trait (Fig 2, Fig. S1c). Our optimal model for fig colour explained 34% of the deviance after controlling for phylogenetic auto-correlation and retained just fig placement and plant maximum size (Table 1). Relative to axial figs, cauliflorous type (i), cauliflorous type (ii) and geocarpic figs, and those on smaller trees were significantly more likely to ripen red.

**Figure 3:**
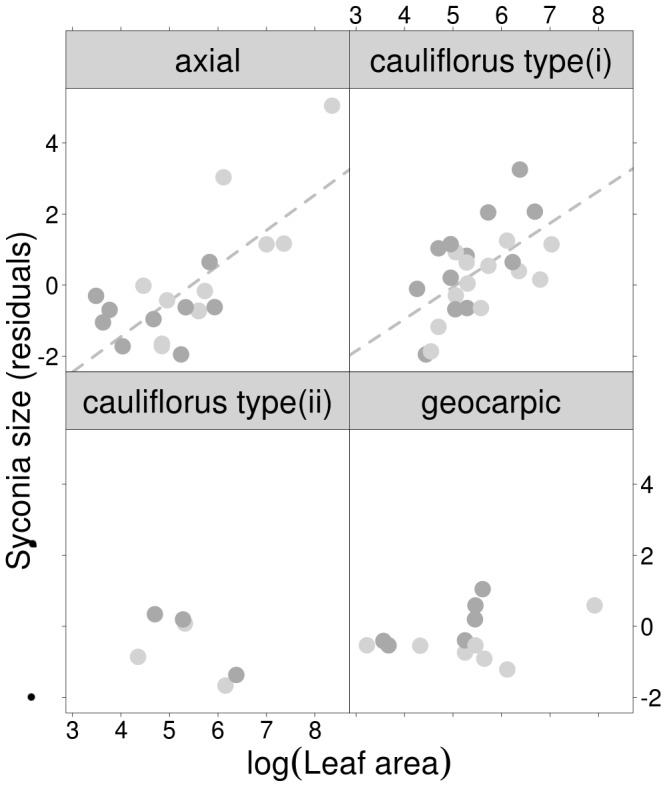
Panel plot of fig size (log transformed) against leaf area (log transformed) by fig placement type. The y-axis represents the residuals after controlling for phylogenetic auto-correlation (see Methods). Dark grey and light grey points represent species with “red” and “green” mature figs, respectively. Relative to axial figs, cauliflorus type (ii) (est = 8.468±3.2355, t = 2.617, *p* = 0.01147) and geocarpic (est = 4.237±1.5943, t = 2.658, *p* = 0.01033) figs were significantly larger, figs on species with larger leaves were significantly larger (est = 0.998±0.1843, t = 5.418, *p* = 0.000001), and there was a significant negative interaction between fig placement and leaf area for cauliflorous type (i) (est = –1.665±0.5972, t = –2.789, *p* = 0.00729) and geocarpic (est = −0.816±0.2978, t = –2.739, *p* = 0.00832) species (*Table S3*).

Fig size varied substantially within lineages (Fig. 2, *Fig S1d*). The optimal model for fig size retained fig placement and leaf area and their interactive effect (Fig. 3; *Table S2*). Relative to axial figs, cauliflorous type (ii) and geocarpic figs were significantly larger, and there was a significant positive effect of leaf size on fig size for axial and cauliflorous type (i) figs.

**Table 1:** Model results of the analysis of fig colour.

Term	Estimate	Std. Error	*t*-value	*P*
Cauliflorous (type i)	2.908	1.0208	2.849	0.004385
Cauliflorous (type ii)	5.593	1.8759	2.982	0.002866
Geocarpic	5.135	1.4713	3.490	0.000483
Plant maximum height	−0.764	0.3511	−2.177	0.029511

Colour was treated as a binomial response (green = 0, red = 1). We controlled for phylogenetic auto-correlation using Moran’s eigenvectors as covariates (not shown for clarity). Variables included in the analysis were fig placement, breeding system (monoecious, dioecious) and biogeographic region as factors, and fig size (log transformed), plant maximum height (square-root transformed), and maximum leaf area (log transformed) as variates. The optimal model retained just plant max. height and fig placement (Deviance explained = 27.8, Residual deviance = 53.6 on 56 d.f). The factor levels for fig placement are compared with axial figs.

As with the other traits, fig placement was an evolutionarily liable trait (Fig. 2, *Fig. S1b*). When we modelled fig placement as a function of the other traits, we found only fig colour and plant size were retained in the optimal model (McFadden r^2^ = 0.407, likelihood ratio test: χ^2^ = 65.7, *P* = 1.69 ×10^–6^, *Table S3*), which was a similar result to that obtained for fig colour.

### Frugivores

Frugivory records were only available for 32 out of the 63 species included in our phylogeny [16], precluding explicit phylogenetic analyses. However, three conclusions can be drawn (Table 2). (i) For all fig placement types, except geocarpic, mixed assemblages of birds or primates and bats comprised the largest category. (ii) Birds have been recorded at all four types of fig placement. (iii) Bats have been recorded at all types of fig placement except geocarpic.

**Table 2:** Frequency table of frugivory records for 32 *Ficus* subgenus *Sycomorus* species.

Fig placement	Frugivory type			
	(a)	(b)	(c)	(d)
Axial	2	0	0	3
Cauliflorous (type i)	1	3	1	9
Cauliflorous (type ii)	0	0	1	5
Geocarpic	2	0	5	0

(a) bird and primate only, (b) bat only, (c) other mammals, and (d) mixed (bat plus bird and/or primate), against fig placement. Data from [16].

## Discussion

Our phylogenetic analysis of *Ficus* subgenus *Sycomorus* produced a tree that was, for the most part, well resolved, and both ML and Bayesian analyses produced similar topologies (Fig. 2). These results provide limited support of the current taxonomy of *Sycomorus* based on morphology (Fig. 2) [17], [18], no doubt reflecting the wide diversity of ecologies and trait convergence (Fig 1 and Fig. 2). Our phylogeny confirmed results of earlier studies in suggesting that the ancestor of *Sycomorus* species was dioecious (*Fig. S1a*) [11].

We found that *Sycomorus* fruit traits (fig placement, size, colour) have been evolutionarily liable (Fig. 2). This is perhaps not surprising as several dioecious species display considerable sexual dimorphism. For example, in cauliflorous species male figs are often borne in large clusters around the base of the tree (Fig 1e), whereas on female trees figs are distributed in smaller clusters along the trunk and main branches [19]. Similarly, among some geocarpic species male figs may be found buried in the soil (Fig. 1h), whereas female figs lie on the soil surface or are suspended from the base of the trunk (Fig 1g).

We examined the association between fruit traits and breeding system, biogeography, and life-history traits. Fruit traits were not significantly associated with either breeding system or biogeography. Thus, neither breeding system nor exposure to different, unrelated lineages of frugivores appears to have constrained the evolution of fruit traits in these fig species. Several of the fig species in our study have wide ranges that extend across Wallace’s line, emphasising the fact that fruit with similar functional attributes can be effectively dispersed by substantially different frugivore assembalges. Fig colour was significantly associated with fig placement and plant maximum size (Table 1), and fig size was significantly associated with fig placement and leaf area (Fig. 3). The fact that smaller trees were more likely to have red figs possibly reflects a preponderance of small frugivorous birds in forest gaps and the understorey [1]. Moreover, it is not unusual to find a correlation between the size of axial fruit and leaf size, as carbon may be allocated directly from proximal leaves [20], [21]. Plant water economy may also play a role, as *Ficus* species use evaporative cooling for both figs and leaves [22]. Hence, dryer environments could simultaneously select for smaller leaves and smaller fruit.

Both fig colour and size were evolutionarily correlated with fig placement, suggesting a central role of fig placement in determining fruit traits in these species. It is often assumed that cauliflory is associated with bat dispersal (e.g. [2]). However, contrary to these expectations, cauliflorous figs were significantly more likely to be red (Table 1) and the figs of most cauliflorous species are fed on by mixed assemblages of frugivores (Table 2). Other observations suggest that cauliflory may not be closely linked to bat dispersal. For example, *F. benguetensis* is a bat-dispersed species with yellow-green figs at maturity. It has cauliflorous figs on male trees that are borne in the typical fashion at the base of the trunk, but axial figs on female trees – suggesting axial placement may sometimes be preferred by bats [19]. Cauliflory may have arisen in response to factors unrelated to frugivory. For example, the production of multiple crops a year, a common trait among cauliflorous species [23], [24], may have resulted in a need to decouple leaf and fig production physiologically and thus lead to fig production on older nodes. Alternatively, it may be linked to the recovery of nutrients from male figs after the pollinating wasps have emerged [19]. Cauliflorous trees drop the male figs closer to the trunk, where nutrients are more likely to be recovered, and in those species with large clusters of male figs at the base of the trunk the branchlets often support a mass of decomposing older figs and have adventitious roots (Fig. 1e). Whether or not these speculations are correct, the common assumption that cauliflory is an evolutionary response to bat dispersal requires reassessment.

Geocarpy arose several times within *Sycomorus* (Fig. 2), although it is comparatively rare among angiosperms as a whole [25]. Our results indicate that geocarpic figs are more likely to be red and larger than axial figs. However, against a background of fallen leaves red is a cryptic colour and it is noteworthy that among these species the immature figs are also red (R. D. Harrison, *personal observations*). In Borneo, geocarpic figs are eaten by small terrestrial mammals, in particular mouse-deer [5], and odour is probably an important cue. These frugivores may provide a relatively efficient seed dispersal service over short distances [1], but it is not clear why the production of long stolons (up to 10 m) or burying figs underground should be advantageous.

Geocarpic figs are early successional pioneer plants and in natural forest are abundant in tree-fall and landslide gaps [5]. In such environments, favourable colonisation microsites often exist close by and geocarpy may enable seeds to germinate directly from where they are produced. In addition, stem mortality is high and most geocarpic figs can regenerate from their stolons when the main stem dies [5], [6]. An association with unstable habitats was the only commonality among a taxonomically broader analysis of geocarpic plants in Africa [25]. Another, mutually compatible, possibility is that invertebrate predators in the leaf litter and soil may limit the numbers of non-pollinating fig wasps. The fact that in those geocarpic species that exhibit sexual dimorphism, it is the male figs that are found buried in the soil supports this conjecture (Fig. 1g & h). Non-pollinators are often super-abundant on cauliflorous species and have a substantial negative impact on pollinator production and thus on pollen dispersal (Fig. 1i) [8]. Thus, we suggest that evolutionary selection for producing fig fruit on long stolons and burying them in leaf litter or soil was probably not driven by seed dispersal agents.

In a study of a wider taxonomic sample of *Ficus* from a single locality in Papua New Guinea, fruit traits were found to be evolutionarily correlated with seed dispersal mode and the authors argued this supported a co-evolutionary hypothesis [2]. However, their study did not investigate correlations with other factors [2]. Moreover, we found that fruit traits were highly liable within *Sycomorus* calling into question the validity of studies, such as this, based on a very small sample of species from a wide taxonomic range. Nevertheless, fruit traits unquestionably have an effect on the frequency of frugivory by different dispersal agents and the colour changes and volatile odours released upon fig ripening almost certainly function as signals to dispersal agents [5], [26]. However, many fig species, including the *Sycomorus* species studied here (Table 2), have mixed assemblages of frugivores [16] and the association between fruit traits and dispersal mode is loose [1]. Although the authors did not draw attention to it, in the study from Papua New Guinea [2] both bat-only and mixed-assemblage dispersed figs occupied almost the entire extent of the multivariate fruit-trait space measured. We also show that among *Sycomorus* species selection for the same fruit traits has occurred in areas with substantially different assemblages of dispersal agents. Thus, caution should be applied in interpreting fruit traits as a co-evolutionary outcome of interactions with a particular group of frugivores. Fruit colour and odour are “broad spectrum” signals that may elicit responses from a wide taxonomic range of potential seed dispersers. Moreover, in *Sycomorus*, we show that plant life-history, both directly and through its effect in determining fig placement, has likely substantially constrained the evolution of fruit traits.

### Conclusions

In *Ficus* subgenus *Sycomorus* fig fruit colour, size and placement were highly evolutionarily liable and similar fruit traits have evolved in regions with substantially different frugivore assemblages. Fig fruit colour and size were significantly associated with plant life-history traits (plant maximum size and leaf area, respectively) and fig placement, after controlling for phylogenetic autocorrelation. In addition, we argue that other aspects of plant ecology, such as phenology, nutrient status, and habitat preference, have been important in constraining fig fruit placement. Thus, relative to plant-life history and other aspects of their ecology, we suggest that the role of dispersal agents may have been comparatively minor in determining fruit traits in these figs.

## Materials and Methods

### Phylogenetic Analyses

Our sampling comprised 63 *Sycomorus* species (global diversity = 130 species) and represents all sections and subsections (*Table S1*). Three species belonging to section *Pharmacosycea* were included as an outgroup [11]. We inferred phylogenetic relationships using five nuclear markers (ITS, 844 bp; ETS, 486 bp; *G3pdh*, 748 bp; *ncpGS*, 1310 bp; *GBSSI* or *Waxy* region, 1673 bp). Extraction, amplification and sequencing protocols followed Rønsted *et al.* [27] (ITS, 844 bp; ETS, 486 bp; *G3pdh*, 748 bp), Silvieus *et al.* [28] (*Waxy*), and Emshwiller and Doyle [29] (*ncpGS*). All sequences have been deposited in GenBank (*Table S1*). Sequence alignment was performed using ClustalW 1.81 default settings [30] followed by a manual adjustment. Phylogenetic trees were estimated using maximum likelihood (ML) and Bayesian methods. The most appropriate model of evolution for each gene was chosen according to AIC values using MrAIC.pl 1.4.3 [31]. Models chosen for each partition were: HKY + Γ (ETS and *Waxy*) and GTR + Γ (ITS, *G3pdh*, *ncpGS*). We performed ML analyses and associated bootstrapping using the MPI-parallelized RAxML 7.0.4 [32]. GTRCAT approximation of models was used for ML boostrapping (1000 replicates). RAxML 7.0.4 does not implement a HKY model, so we used GTR instead. We used a discrete gamma distribution with four categories. Bayesian analyses were conducted using a parallel version of MrBayes v. 3.1.1. [33]. We assumed across-partition heterogeneity in model parameters by considering the parameter m. Parameter values for the model were initiated with default uniform priors and branch lengths were estimated using default exponential priors. To improve mixing of the cold chain and avoid it converging on local optima, we used Metropolis-coupled Markov Chain Monte Carlo (MCMCMC) with each run including a cold chain and three incrementally heated chains. The heating parameter was set to 0.02 in order to allow swap frequencies from 20 to 70% [33]. We ran two independent runs of 30 million generations. All values were sampled every 3000 generations. For the initial determination of burn-in, we examined the plot of overall model likelihood against generation number to find the point where the likelihood started to fluctuate around a constant value. The points sampled prior to convergence of the chains were then discarded. We used a range of MCMC convergence and good mixing diagnostics following Cruaud et al. [34] and all Bayesian searches showed evidence of sufficiently long burn-ins and convergence on the stationary distribution (eg ESS and avstdeviation of split frequencies). The results were based on the pooled samples from the stationary phases of the two independent runs.

### Functional Traits

Among plant functional traits, we studied breeding system, fruit traits (placement, size, colour), leaf area and maximum plant height. These traits are important determinants of plant reproductive strategy, seed dispersal mode, and plant life-history [16], [35]. Data were obtained from recent taxonomic accounts [17], [18], augmented with field observations. For dioecious species, we used female characters as we were interested in the evolution of seed dispersal mode.

Fig placement was classified as: axial (Fig. 1b); cauliflorous with figs borne on woody nodes or branchlets (Fig. 1a and d, cauliflorous type (i)); cauliflorous with long rope-like stolons (Fig. 1f, cauliflorous type (ii)); and geocarpic (Fig. 1g and h). For fig size, we used reported diameter measurements and used data for maximum dry diameter, as measured on herbarium material. Smaller diameters often represent immature figs and the fresh diameters given in taxonomic accounts are estimated and rarely reliable. Mature fruit colours were divided into two groups; pink-red-purple-black (hereafter “red”) and green-yellow-brown (hereafter “green”). Red figs usually mature pink-to-reddish initially and gradually turn purple or black. Purple and black fruit often reflect strongly in the violet to UV spectrum. Red fruit contrast strongly with foliage or bark [16]. It is important to bear in mind that fruit colour interacts with the visual sensitivity of potential frugivores. Old World apes and monkeys are trichromats, with good red vision, but most other mammals are dichromats; birds are trichromats with increased sensitivity to violet or UV wavelengths; whereas Old World fruit bats include both dichromats and monochromats [37]. Dichromats can often distinguish shades of green better than trichromats, but may not be able to distinguish red or black fruit from foliage. Thus, red figs should be conspicuous to apes, monkeys and birds, but will be inconspicuous to most other mammals, including bats. Meanwhile, green figs will be visually inconspicuous to apes, monkeys and birds, but conspicuous to most other mammals, including some bats. Green figs also invariably produce odours. As most birds are trichromats with a poor sense of smell, being green tends to exclude birds [16]. Some red figs also produce odours and thus may be conspicuous to almost all potential frugivores [16]. We estimated leaf area as an oval (π*½ width*½ length) from the reported maximum leaf length (not including drip-tip) and maximum leaf width. This slightly under-estimates the area of leaves with parallel sides, but such errors will be small compared to the overall variation in leave size.

### Frugivores

Data on frugivores were obtained from the global review of frugivory at figs [16]. Data were available for only 32 of the 63 species included in our study. Also, many of the surveys included in this review were of short duration and hence the data should be considered “presence only” records (i.e. lack of a recorded frugivore interaction cannot be taken to mean it does not exist).

### Data Analyses

We modelled fig colour, size and placement as a function of the other five traits and biogeographic distribution. We classified biogeographic distributions into three categories: Africa (+Madagascar-India Ocean), Asia (west of Wallace line), and Pacific (east of Wallace line). To control for phylogenetic auto-correlation, we calculated Moran’s eigenvectors based on Abouheif distances across the tips of the ML phylogeny (function ‘me.phylo’ in R package ‘adephylo’ [36], [37]), and then in each model we sequentially incorporated the largest eigenvectors as covariates until no significant phylogenetic auto-correlation remained among the residuals (function ‘orthogram’). To avoid over-saturating models, addition and removal of terms was conducted manually until we arrived at the optimal model according to AIC values. Fig colour was treated as binomial response (function ‘glm’, family = binomial, link = logit), placement as a multinomial response (function ‘mlogit’, package ‘mlogit’), and size as a normal response (function ‘lm’). Fig size and leaf area were log transformed and plant height was square-root transformed. All analyses were conducted in R v2.14.1 [38].

## Supporting Information

Figure S1 Phylogeny of *Ficus* subgenus *Sycomorus* in relation to (a) breeding system, (b) fig placement, (c) fig colour, and (d) fig size (dry diameter). For (a), (b) and (c) the proportional likelihood of each state is mapped across nodes. For (d) the predicted size is mapped across nodes. Ancestral reconstructions were conducted using maximum likelihood as implemented in ‘ace’ from the R package ‘ape’. This figure is available as a separate file.
(TIFF)

Table S1 Species of *Ficus* used for phylogenetic analysis, associated accession numbers of ITS, ETS, *G3pdh, ncpGS, waxy* region and sequence origin.
(DOC)

Table S2 Results of the analysis of fig size (diameter). Fig size (log transformed) was modeled with Gaussian errors. We controlled for phylogenetic auto-correlation using Moran’s eigenvectors as covariates (not shown for clarity). Variables included in the analysis were fig placement, fig colour, breeding system (monoecious, dioecious) and biogeographic region as factors, and plant maximum height (square-root transformed), and maximum leaf area (log transformed) as variates. The model with the lowest AIC retained fig placement, leaf area and their interaction (adjusted r^2^ = 0.453, F_8,54_ = 7.42, *P* = 1.306×10^–6^).
(DOC)

Table S3 Results of the analysis of fig placement. Fig placement was treated as a multinomial response. We controlled for phylogenetic auto-correlation using Moran’s eigenvectors as covariates (not shown for clarity). Variables included in the analysis were fig colour, breeding system (monoecious, dioecious), and biogeographic region as factors, and fig size (log transformed), plant maximum height (square-root transformed), and leaf area (log transformed) as variates. The optimal model retained just plant max. height and fig colour (McFadden r^2^ = 0.407, likelihood ratio test: χ^2^ = 65.7, *P* = 1.69×10^–6^).
(DOC)
