# Supplementary figures and images for: Evolution of Fruit Traits in Ficus Subgenus Sycomorus (Moraceae): To What Extent Do Frugivores Determine Seed Dispersal Mode?
Source: PLoS One. 2012 Jun 5;7(6):e38432. doi: 10.1371/journal.pone.0038432 (PMC3367955; doi:10.1371/journal.pone.0038432)

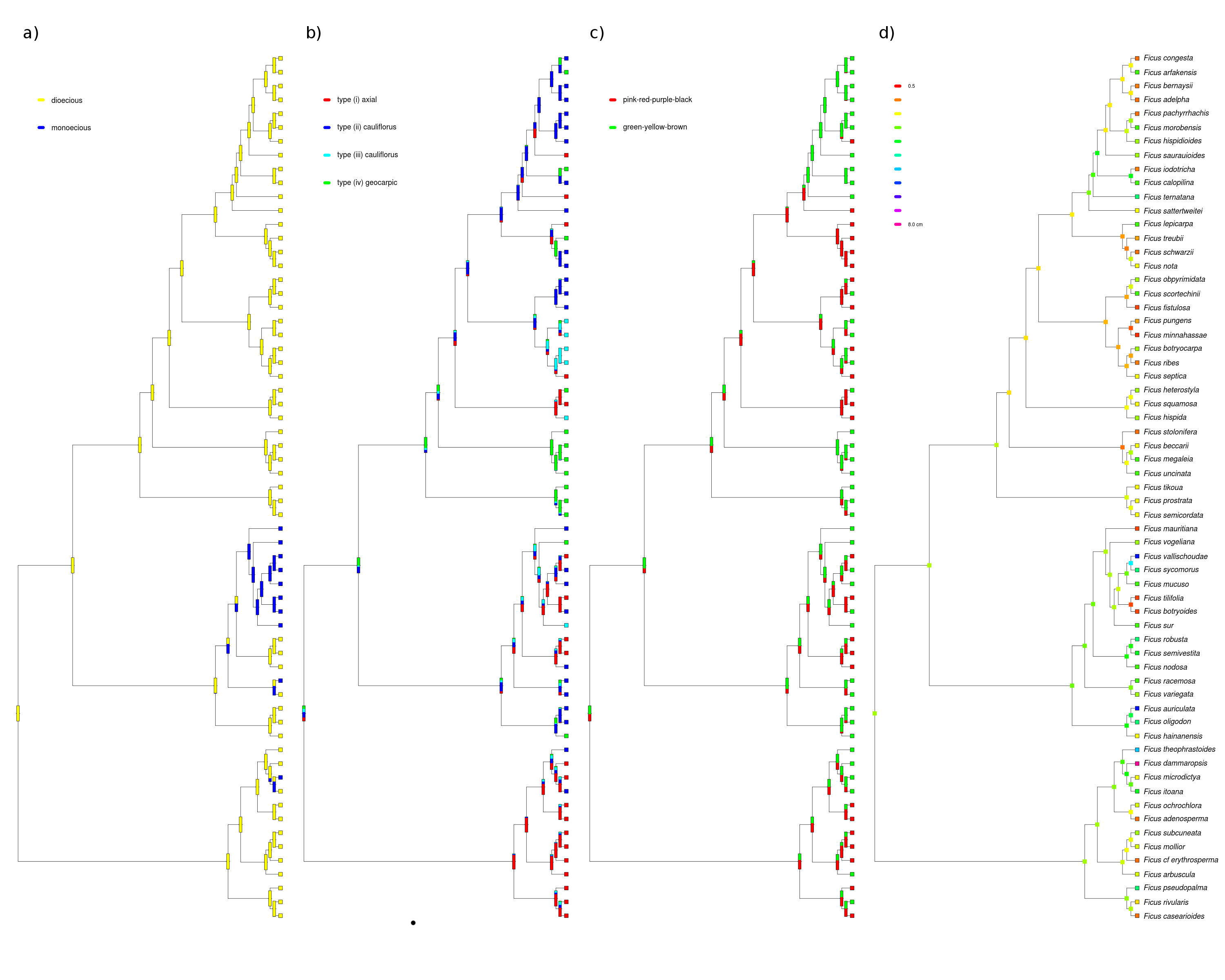

Supplement: Figure S1 — Phylogeny of Ficus subgenus Sycomorus in relation to (a) breeding system, (b) fig placement, (c) fig colour, and (d) fig size (dry diameter). For (a), (b) and (c) the proportional likelihood of each state is mapped across nodes. For (d) the predicted size is mapped across nodes. Ancestral reconstructions were conducted using maximum likelihood as implemented in ‘ace’ from the R package ‘ape’. This figure is available as a separate file. (TIFF) [file pone.0038432.s001.tiff]
