# Supplementary material for: Evolution of Fruit Traits in Ficus Subgenus Sycomorus (Moraceae): To What Extent Do Frugivores Determine Seed Dispersal Mode?
Source: PLoS One. 2012 Jun 5;7(6):e38432. doi: 10.1371/journal.pone.0038432 (PMC3367955; doi:10.1371/journal.pone.0038432)
Supplement: Table S1 — Species of Ficus used for phylogenetic analysis, associated accession numbers of ITS, ETS, G3pdh, ncpGS, waxy region and sequence origin. (DOC) [file pone.0038432.s002.doc]

**Table S1.**

| Section | Subsection | Species | GenBank accession number |
| --- | --- | --- | --- |
|  |  |  | ITS/ETS/G3pdh/*ncpGS*/ *waxy* |
| *Adenosperma* |  | *F. adenosperma* Miq. | AF165374/EF092321/EF092374/EU084324/DQ367636 |
|  |  | *F. arbuscula* Laut. & K. Schum. | EU091617/-/-/-/EU084375 |
|  |  | *F. casearioides* King*.* | EU091618/-/EU087654/-/- |
|  |  | *F.* cf*. erythrosperma* Miq*.* | DQ457093/-/DQ457092/-/- |
|  |  | *F. mollior* Benth. | DQ367658/-/DQ367623/-/DQ367643 |
|  |  | *F. subcuneata* Miq. | EU091620/-/DQ367631/-/DQ367651 |
| *Bosscheria* |  | *F. minnahassae* (Teijs/Vrie) Miq | -/-/EU087662/-/EU084380 |
|  |  | *F. pungens* Blume | -/-/DQ367627/-/DQ367647 |
| *Dammaropsis* |  | *F. dammaropsis* Diels | AF165383/ EU084445/DQ367621/-/ DQ367641 |
|  |  | *F. pseudopalma* Blanco | EU091629/-/ EU087664/EU084329/- |
|  |  | *F. rivularis* Merr*.* | EU091619/-/ EU087657/-/- |
|  |  | *F. theophrastoides* Seem*.* | AF165412/-/-/-/- |
| *Hemicardia* |  | *F. prostrata* Miq*.* | EU091612/JN117668/-JN117708/-/- |
|  |  | *F. semicordata* J.E. Smith | JN117646 /-/JN117710 /EU084322/- |
|  |  | *F. tikoua* Bureau | EU091641/JN117679/EU087673/-/- |
| *Papuasyce* |  | *F. itoana* Diels | AF165391/-/EU087655/-/EU084376 |
|  |  | *F. microdictya* Diels | AF165394/ EU084447/ EU087656/-/ EU084377 |
| *Sycomorus* | *Neomorphe* | *F. auriculata* Lour*.* | AF165376/ FJ812281/ EU087653/-/ EU084374 |
|  |  | *F. hainanensis* Merr. & Chun | EU091614/-/JN117697/-/- |
|  |  | *F. nodosa* Teysm.et. Binn. | AF165395/-/DQ367625/-/DQ367645 |
|  |  | *F. oligodon* Miq. | JN117641/-/JN117706-/- |
|  |  | *F. robusta* Corner | AF165406/-/DQ367628/-/DQ367648 |
|  |  | *F. semivestita* Corner | -/EU084443/DQ367629/-/DQ367649 |
|  |  | *F. variegata* King | -/-/-/-/DQ367653 |
|  | *Sycomorus* | *F. botryoides* Baker | AF165380/-/-/-/- |
|  |  | *F. mauritiana* Lam*.* | AY063570/-/EF092371/-/- |
|  |  | *F. mucuso* Ficalho*.* | AY730120/AY730210/EF092372/EU084317/- |
|  |  | *F. racemosa* Linn. | -/-/JN126051/EU084318/EU084371 |
|  |  | *F. sur* Forssk*.* | AY063572/-/EU087649/EU084319/EU084372 |
|  |  | *F. sycomorus* Linn*.* | AY063575/AY063536/-/EU084320/- |
|  |  | *F. tiliifolia* Baker | EU091609/EU084439/-/-/- |
|  |  | *F. vallis-choudae* Delile*.* | AY063574/-/-/EU084321/EU084373 |
|  |  | *F. vogeliana* (Miq.) Miq. | EU091610/EU084440/EU087650/-/- |
| *Sycocarpus* | *Macrostyla* | *F. squamosa* Roxb*.* | EU091634/-/-/-/- |
|  | *Sycocarpus* | *F. adelpha* Laut. & K. Schum. | DQ367656/-/DQ367615/-/- |
|  |  | *F. arfakensis* King*.* | DQ367657/-/DQ367617/-/DQ36737 |
|  |  | *F. beccarii* King | EU091621/-/EU087658/-/- |
|  |  | *F. bernaysii* King*.* | AF165378/-/DQ367618/-/DQ367638 |
|  |  | *F. botryocarpa* Miq. | AF165379/-/ DQ367619/-/DQ367639 |
|  |  | *F. calopilina* Diels | EU091622/-/-/-/- |
|  |  | *F. ternatana* (Miq.) Miq. | DQ367662/-/ DQ367632/-/DQ367652 |
|  |  | *F. congesta* Roxb*.* | AY730136/-/-/-/DQ367640 |
|  |  | *F. fistulosa* Reinw. ex. Blume | AY730137/-/EF092375/-/EU084379 |
|  |  | *F. heterostyla* Merr*.* | EU091611/-/EU087651/-/- |
|  |  | *F. hispida* Linn. | EU091623/-/JN117700/EU084326/- |
|  |  | *F. hispidoides* S. Moore. | AF165388/-/DQ367622/-/DQ367642 |
|  |  | *F. iodotricha* Diels | EU091624/-/EU087660/-/- |
|  |  | *F. lepicarpa* Blume | AY730138/-/EF092376/-/- |
|  |  | *F. megaleia* Corner | EU091625/-/EU087661/-/- |
|  |  | *F. morobensis* C.C. Berg | DQ367659/-/DQ367624/-/DQ367644 |
|  |  | *F. nota* (Blanco) Merr. | EU091626/-/ EU087663/EU084327/- |
|  |  | *F. obpyrimidata* King | EU091627/-/-/-/- |
|  |  | *F. pachyrrhachis* Laut. & K. Schum | EU091628/-/DQ367626/EU084328/DQ367646 |
|  |  | *F. ribes* Reinw. ex Blume | EU091630/-/EU091630/-/- |
|  |  | *F. sattherthwaitii* Elmer | EU091631/-/EU087666/-/- |
|  |  | *F. saurauroides* Diels | EU091632/-/EU087667/-/EU084381 |
|  |  | *F. schwarzii* Koord*.* | EU091633/-/-/-/- |
|  |  | *F. scortechinii* King*.* | AY730139/-/EF092377/-/- |
|  |  | *F. septica* Burm. F. | AF165409/-/DQ367630/-/DQ367650 |
|  |  | *F. stolonifera* King | EU091635 |
|  |  | *F. treubii* King | EU091636/-/EU087668/-/- |
|  |  | *F. uncinata* King*.* | AY063576/-/EU087669/-/- |
|  |  | *F. ochrochlora* Ridley | AF165396/-/-/-/EU084378 |
| *Pharmacosycea* | *Bergianae* | *F. yoponensis* Desvaux. | AY063594/AY063552/AY967959/-/- |
|  | *Petenenses* | *F. maxima* Miller | AY063595/AY063551/AY967958/-/- |
|  |  | *F. tonduzii* Standl. | AY730140/AY730230/EU087611/EU084297/ |
