# Supplementary material for: Evolution of Fruit Traits in Ficus Subgenus Sycomorus (Moraceae): To What Extent Do Frugivores Determine Seed Dispersal Mode?
Source: PLoS One. 2012 Jun 5;7(6):e38432. doi: 10.1371/journal.pone.0038432 (PMC3367955; doi:10.1371/journal.pone.0038432)
Supplement: Table S2 — Results of the analysis of fig size (diameter). Fig size (log transformed) was modeled with Gaussian errors. We controlled for phylogenetic auto-correlation using Moran’s eigenvectors as covariates (not shown for clarity). Variables included in the analysis were fig placement, fig colour, breeding system (monoecious, dioecious) and biogeographic region as factors, and plant maximum height (square-root transformed), and maximum leaf area (log transformed) as variates. The model with the lowest AIC retained fig placement, leaf area and their interaction (adjusted r2 = 0.453, F8,54 = 7.42, P = 1.306×10^–6^). (DOC) [file pone.0038432.s003.doc]

| **Table S2.** | | | | |
| --- | --- | --- | --- | --- |
| Term | Estimate | Std. Error | *t*-value | *P* |
| Cauliflorus type (i) | 0.888 | 1.7864 | 0.497 | 0.621070 |
| Cauliflorus type (ii) | 8.468 | 3.2355 | 2.617 | 0.011470 |
| Geocarpic | 4.237 | 1.5943 | 2.658 | 0.010330 |
| Leaf area | 0.998 | 0.1843 | 5.418 | 0.000001 |
| Cauliflorus type (i): leaf area | -0.099 | 0.3246 | -0.306 | 0.760740 |
| Cauliflorus type (ii): leaf area | -1.665 | 0.5972 | -2.789 | 0.007290 |
| Geocarpic: leaf area | -0.816 | 0.2978 | -2.739 | 0.008320 |
