# Supplementary material for: Evolution of Fruit Traits in Ficus Subgenus Sycomorus (Moraceae): To What Extent Do Frugivores Determine Seed Dispersal Mode?
Source: PLoS One. 2012 Jun 5;7(6):e38432. doi: 10.1371/journal.pone.0038432 (PMC3367955; doi:10.1371/journal.pone.0038432)
Supplement: Table S3 — Results of the analysis of fig placement. Fig placement was treated as a multinomial response. We controlled for phylogenetic auto-correlation using Moran’s eigenvectors as covariates (not shown for clarity). Variables included in the analysis were fig colour, breeding system (monoecious, dioecious), and biogeographic region as factors, and fig size (log transformed), plant maximum height (square-root transformed), and leaf area (log transformed) as variates. The optimal model retained just plant max. height and fig colour (McFadden r2 = 0.407, likelihood ratio test: χ2 = 65.7, P = 1.69×10^–6^). (DOC) [file pone.0038432.s004.doc]

| **Table S3.** | | | | |
| --- | --- | --- | --- | --- |
| Term | Estimate | Std. Error | *t*-value | *P* |
| Cauliflorus (type i): Plant height | 0.674 | 0.3800 | 1.773 | 0.076235 |
| Cauliflorus (type ii): Plant height | 2.735 | 0.8864 | 1.994 | 0.046150 |
| Geocarpic: Plant height | 0.252 | 0.4299 | 0.585 | 0.560000 |
| Cauliflorus (type I): Colour | 2.735 | 1.0261 | 2.665 | 0.007695 |
| Cauliflorus (type ii): Colour | 3.429 | 1.8537 | 1.850 | 0.064310 |
| Geocarpic: Colour | 6.790 | 2.3571 | 2.881 | 0.003970 |
